# Two optimized novel potential formulas and numerical algorithms for $m\times n$ cobweb and fan resistor networks

**DOI:** 10.1038/s41598-023-39478-8

**Published:** 2023-07-31

**Authors:** Wenjie Zhao, Yanpeng Zheng, Xiaoyu Jiang, Zhaolin Jiang

**Affiliations:** 1 School of Automation and Electrical Engineering, Linyi University, Linyi, 276000 China; 2 School of Information Science and Engineering, Linyi University, Linyi, 276000 China; 3 School of Mathematics and Statistics, Linyi University, Linyi, 276000 China

**Keywords:** Mathematics and computing, Physics

## Abstract

The research of resistive network will become the basis of many fields. At present, many exact potential formulas of some complex resistor networks have been obtained. Computer numerical simulation is the trend of computing, but written calculation will limit the time and scale. In this paper, the potential formulas of a $m\times n$ scale cobweb resistor network and fan resistor network are optimized. Chebyshev polynomial of the second class and the absolute value function are used to express the novel potential formulas of the resistor network, and described in detail the derivation process of the explicit formula. Considering the influence of parameters on the potential formulas, several idiosyncratic potential formulas are proposed, and the corresponding three-dimensional dynamic images are drawn. Two numerical algorithms of the computing potential are presented by using the mathematical model and *DST*-*VI*. Finally, the efficiency of calculating potential by different methods are compared. The advantages of new potential formulas and numerical algorithms by the calculation efficiency of the three methods are shown. The optimized potential formulas and the presented numerical algorithms provide a powerful tool for the field of science and engineering.

## Introduction

Tan^1^ creatively established the mathematical model of cobweb and fan resistor networks, according to this model, gave the incomparable analytical potential formula in theory. This is a breakthrough work, and its theoretical significance and application prospects are huge. As is well-known, classical physics is based on the analysis of physics mathematical models of physical processes. Computers have given physicists and engineers a new way to analyze and apply physical formulas and mathematical models that has revolutionized science and engineering outside the university. Everything changes if the computer is used to analyze and apply physical formulas and mathematical models. In addition, experts in engineering and scientific computing know that to improve the computational efficiency of the potential to help computational physicists and engineers solve major scientific and technical problems, it is a good idea to optimize the perfect analytical potential formula given in theory to improve the computational efficiency. In order to improve the calculation performance and scale of the formula. In this paper, based on the original potential formula, we re-represent it with the Chebyshev polynomial of the second class and the absolute value function, which improves the computational efficiency, and design the numerical algorithms can be used to the calculating potential for large-scale resistor networks.

In the process of scientific development, many complex problems have arisen, which often require simple models to solve. According to the research results of resistor network model^2–11^ and neural network model^12–18^, ideas can be obtained on many complex problems. In the past many years, through the research results of Green’s function, Laplace equation, Poisson equation, finite and infinite dimensional resistor network and Laplace matrix (*LM*) method and so on^8–11,19–32^, the foundation of resistor network research has been laid. Shi et al.^12,13^ studied a new discrete time recurrent neural network and its application to manipulators. Sun et al.^14,15^ studied the theory and application of noise tolerance zeroing neural network. And Jin et al.^16–18^ proposed a modified Zhang neural network (*MZNN*) model for the solution of time-varying quadratic programing (*TVQP*).

In the past few years, Tan et al.^33–53^ proposed a simpler recursive transformation (*RT*) method than *LM* method in the research of resistance network. It simplified the Laplacian matrix in two directions to the Laplacian matrix in one direction. In 2014, Tan et al.^37^ solved the potential formula of spherical resistance network for the first time. Since 2015, Tan et al.^34–44^ has studied the resistance network model by *RT* method. After 2020, Tan et al.^45–53^ made more in-depth research on resistance network. Since *RT* method requires using a tridiagonal matrix to construct a mathematical model, and the analytical potential formula must be expressed by using the exact eigenvalues of this tridiagonal matrix. So the exact eigenvalues of the tridiagonal matrix need to be found. Tridiagonal matrices are used in many areas of science and engineering, and there are many good conclusions about it^54–61^.

**Figure 1:**
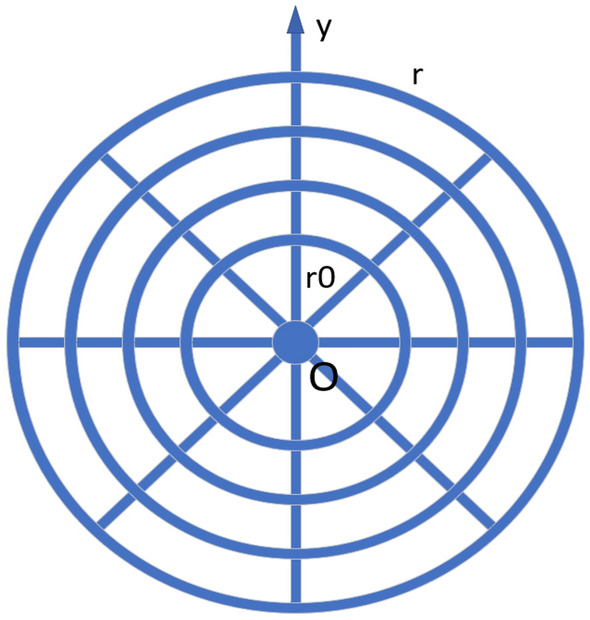
A $8 \times 4$ cowbeb resistor network containing $8 \times 4$ nodes and a zero potential point *O*.

**Figure 2:**
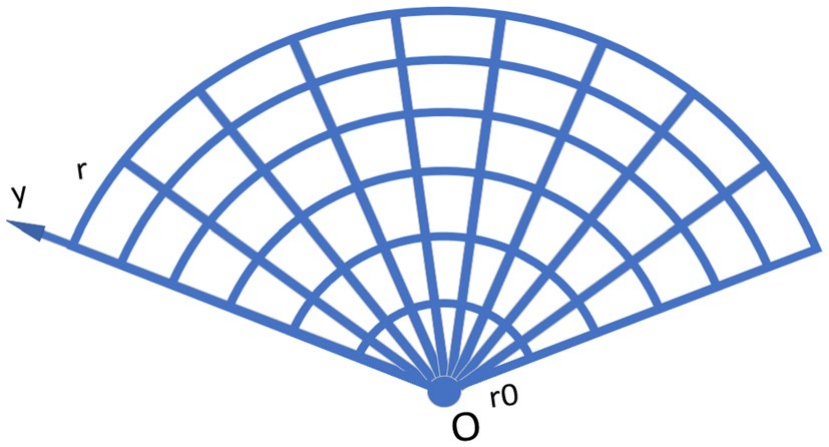
A $10 \times 6$ fan resistor network containing $10 \times 6$ nodes and a zero potential point *O*.

In 2017, Tan^1^ used *RT*-*V* method for the first time to study cobweb network and fan network. In Figs. 1 and 2, the resistance on the warp and weft lines is $r_{0}$ and *r*, where *m* and *n* are the scale of the resistor network, it contains *m* rows and *n* columns. Point $O_{(0,0)}=0$ is defined as the origin of the resistor network. The potential formula $U_{m\times n}(x, y )$ of any node *d*(*x*, *y*) in the $m\times n$ cobweb network is shown as

1 $$\begin{aligned} \frac{U_{m\times n}(x,y)}{J} = \frac{4r}{2m+1}\sum ^{m}_{i=1}\frac{g^{(i)}_{x_{1},x}S_{y_{1},i}-g^{(i)}_{x_{2},x} S_{y_{2},i}}{\lambda ^{n}_{i}+{{\bar{\lambda }}}^{n}_{i}-2}S_{y,i}, \end{aligned}$$

2 $$\begin{aligned} g^{(i)}_{x_{s},x} = F_{n-|x_{s}-x|}+F_{|x_{s}-x|}. \end{aligned}$$

The potential formula $U_{m\times n}(x, y )$ of any node *d*(*x*, *y*) in the $m\times n$ fan network is shown as

3 $$\begin{aligned} \frac{U_{m\times n}(x,y)}{J} = \frac{4r}{2m+1}\sum ^{m}_{i=1}\frac{\beta ^{(i)}_{x_{1},x}S_{y_{1},i} -\beta ^{(i)}_{x_{2},x}S_{y_{2},i}}{(t_{i}-2)F^{(i)}_{n+1}}S_{y,i}, \end{aligned}$$

4 $$\begin{aligned} \beta ^{(i)}_{x_{s},x} = \left\{ \begin{aligned} \Delta F^{(i)}_{x_s}\Delta F^{(i)}_{n-x} ~~if~x\ge x_s,\\ \Delta F^{(i)}_{x}\Delta F^{(i)}_{n-x_s} ~~if~x\le x_s, \end{aligned} \right. \end{aligned}$$

where $$\theta _{i} = \frac{(2i-1)\pi }{(2m+1)},\ S_{y_k,i} = \sin (y_{k}\theta _{i})$$

5 $$\begin{aligned} F^{(i)}_{k} = \frac{\lambda ^{k}_{i}-{\bar{\lambda }}^{k}_{i}}{\lambda _{i}-{\bar{\lambda }}_{i}}, \end{aligned}$$

6 $$\begin{aligned}{}&\lambda _{i} = 1+h-h\cos \theta _{i}+\sqrt{(1+h-h\cos \theta _{i})^{2} - 1},\\&{\bar{\lambda }}_{i} = 1+h-h\cos \theta _{i}-\sqrt{(1+h-h\cos \theta _{i})^{2} - 1}, \end{aligned}$$

7 $$\begin{aligned} t_{i} = 2+2h-2h\cos {\theta _i}, \end{aligned}$$

the parameter $h=\frac{r}{r_0}$ is defined.

## Novel formulas of potential represented by Chebyshev polynomials

This section presents the re-expressed potential formulas (1) and (3)^1^ of the resistor network. The potential formula expressed by the Chebyshev polynomial of the second class^62^ can reduce the running time of computer simulation.

Assume that the current *J* is input from $d_{1}(x_{1}, y_{1})$ and output from $d_{2}(x_{2}, y_{2})$. The potential formula of any node *d*(*x*, *y*) in the $m\times n$ cobweb resistor network is

8 $$\begin{aligned} \frac{{\textbf{U}}_{m\times n}(x,y)}{J} = \frac{4r}{2m+1}\sum ^{m}_{\iota =1}\frac{\mu ^{(\iota )}_{x_{1},x}S_{y_{1},\iota } -\mu ^{(\iota )}_{x_{2},x}S_{y_{2},\iota }}{U^{(\iota )}_{n}-U^{(\iota )}_{n-2}-2}S_{y,\iota }, \end{aligned}$$

where

9 $$\begin{aligned} \mu ^{(\iota )}_{x_{s},x} = U^{(\iota )}_{n-|x_{s}-x|-1}+U^{(\iota )}_{|x_{s}-x|-1},~~ s = 1,~2. \end{aligned}$$

The potential formula of any node *d*(*x*, *y*) in the $m\times n$ fan resistor network is

10 $$\begin{aligned} \frac{{\mathcal {U}}_{m\times n}(x,y)}{J} = \frac{4r}{2m+1}\sum ^{m}_{\iota =1}\frac{\varepsilon ^{(\iota )}_{x_{1},x}S_{y_{1},\iota } -\varepsilon ^{(\iota )}_{x_{2},x}S_{y_{2},\iota }}{(\omega _{\iota }-2)U^{(\iota )}_{n}}S_{y,\iota }, \end{aligned}$$

where

11 $$\begin{aligned} \varepsilon ^{(\iota )}_{x_{s},x}=({U^{(\iota )}_{-0.5}})^2(U^{(\iota )}_{n-|x_s-x|+1} -U^{(\iota )}_{n-|x_s-x|-1}+U^{(\iota )}_{n-x_s-x}-U^{(\iota )}_{n-x_s-x-2}),~~ s = 1,~2, \end{aligned}$$

12 $$\begin{aligned} S_{y_k,\iota } = \sin (\frac{y_k(2\iota -1)\pi }{2m+1}),~~k=1,~2, \end{aligned}$$

13 $$\begin{aligned} \omega _{\iota }=2+\frac{2r}{r_{0}}-\frac{2r}{r_{0}}\cos \frac{(2\iota -1)\pi }{2m+1}, \end{aligned}$$

14 $$\begin{aligned} U^{(\iota )}_{\nu }= U^{(\iota )}_{\nu }(\cosh \phi _{\iota })= \frac{\sinh (\nu +1)\phi _{\iota }}{\sinh (\phi _{\iota })}, \cosh \phi _{\iota }=\frac{\omega _{\iota }}{2},~~\frac{\omega _{\iota }}{2}>1,~~\phi _{\iota }>0, \end{aligned}$$

$$\begin{aligned} v=n-|x_s-x|-1,~|x_s-x|-1,~n-2,~n-|x_s-x|+1,~n-x_s-x,~n-x_s-x-2,~n,~-0.5,~~ s=1,~2,~~\iota =1,~2,\ldots ,~m. \end{aligned}$$

We set the node voltage at point $O_{(0,0)}$ to 0, and the formula for calculating the potential of any node is described as

15 $$\begin{aligned} {\textbf{U}}_{m\times n}(x,y) = {\textbf{V}}^{(y)}_{x},~{\mathcal {U}}_{m\times n}(x,y) = {\mathcal {V}}^{(y)}_{x},~V^{(0)}_{0}=0, \end{aligned}$$

where ${\textbf{V}}^{(y)}_{x}$ and ${\mathcal {V}}^{(y)}_{x}$ are denoted by the node voltage of any node.

## Horadam sequence and discrete sine transform

In this section, we introduce the explicit formula of Horadam sequence which is expressed by the Chebyshev polynomial of the second class and the sixth kind of discrete sine transform.

A second-order recurrence sequence ${W_v}$ is called a Horadam sequence if

16 $$\begin{aligned} W_v=dW_{v-1}-qW_{v-2},~~W_0=a,~~W_1=b, \end{aligned}$$

where $v\in {\textbf{N}},~~v\ge 2,~~a,~b,~d,~q\in {\mathbb {C}}$, ${\textbf{N}}$ is set of all nonnegative integers and ${\mathbb{C}}$ is the set of all complex numbers.

The explicit formula of Horadam sequence expressed by the Chebyshev polynomial of the second class is^63^

17 $$\begin{aligned} W_{v} =(\sqrt{q})^v\left( \frac{b}{\sqrt{q}}U_{v-1}\left( \frac{d}{2\sqrt{q}}\right) - aU_{v-2}\left( \frac{d}{2\sqrt{q}}\right) \right) , \end{aligned}$$

where $U_{v}$ is the Chebyshev polynomial of the second class^62^, i.e.

18 $$\begin{aligned} U_{v}=U_{v}(\cos \phi ) = \frac{\sin (v+1)\phi }{\sin \phi },~~\cos \phi =\frac{d}{2\sqrt{q}},~~\phi \in {\mathbb {C}}. \end{aligned}$$

If $\frac{d}{2\sqrt{q}} >1$, the Chebyshev polynomial of the second class is re-described by hyperbolic functions, then Eq. (18) is transformed into

19 $$\begin{aligned} U_{v}=U_{v}(\cosh \phi ) = \frac{\sinh (v+1)\phi }{\sinh \phi },~~\cosh \phi =\frac{d}{2\sqrt{q}},~~\phi \in {\mathbb {R}}, \end{aligned}$$

where ${\mathbb {R}}$ is the set of all real numbers.

First, we will present the derivation of Eq. (5) represented by the Chebyshev polynomial of the second class.

### Remark 1

It can be obtained from Eq. (6) that $\lambda _{\iota }+{\bar{\lambda }}_{\iota }=\omega _{\iota }$ and $\lambda _{\iota }\cdot {\bar{\lambda }}_{\iota }=1$. Adding these conditions to Eq. (16), we get the following special Horadam sequence

20 $$\begin{aligned} F^{(\iota )}_v=\omega _{\iota }F^{(\iota )}_{v-1}-F^{(\iota )}_{v-2}, ~~F^{(\iota )}_{0}=0,~~F^{(\iota )}_{1}=1, \end{aligned}$$

where $d=\omega _{\iota }>2,~~q=1$, $F^{(\iota )}_{v}$ and $\omega _{\iota }$ are expressed in Eqs. (5) and (13), respectively. By replacing the expression of Eq. (5), with the results of Eq. (19), we have

21 $$\begin{aligned} F^{(\iota )}_v = \frac{ \lambda ^{v}_{\iota }-{\bar{\lambda }}^{v}_{\iota }}{\lambda _{\iota } -{\bar{\lambda }}_{\iota }}=U^{(\iota )}_{v-1}(\frac{\omega _{\iota }}{2}). \end{aligned}$$

Secondly, we will give the derivation of $\lambda ^{n}_{\iota }+{\bar{\lambda }}^{n}_{\iota }$ expressed by the Chebyshev polynomial of the second class.

### Remark 2

Let

22 $$\begin{aligned} B^{(\iota )}_{n}=\lambda ^{n}_{\iota }+{\bar{\lambda }}^{n}_{\iota }, \end{aligned}$$

where $$B^{(\iota )}_{0}=2,\ B^{(\iota )}_{1}=\omega _{\iota }$$.

Then the recursive relation of $B^{(\iota )}_{n}$ is expressed as

23 $$\begin{aligned} B^{(\iota )}_{n}=\omega _{\iota }B^{(\iota )}_{n-1}-B^{(\iota )}_{n-2},~~B^{(\iota )}_{0}=2,~ B^{(\iota )}_{1}=\omega _{\iota }, \end{aligned}$$

where $d=\omega _{\iota },\ q=1$, $\omega _{\iota }$ and $B^{(\iota )}_{n}$ are expressed in Eqs. (13) and (22), respectively.

By Eqs. (17) and (19), $B^{(\iota )}_{n}$ is represented as follows

24 $$\begin{aligned} B^{(\iota )}_{n}=\lambda ^{n}_{\iota }+{\bar{\lambda }}^{n}_{\iota } =\omega _{\iota }U_{n-1}(\frac{\omega _{\iota }}{2})-2U_{n-2}(\frac{\omega _{\iota }}{2}) =U_{n}(\frac{\omega _{\iota }}{2})-U_{n-2}(\frac{\omega _{\iota }}{2}). \end{aligned}$$

Next, we will show the derivation of replacing Eq. (4) in terms of piecewise functions with Eq. (11) in terms of absolute value functions.

### Remark 3

For Eq. (4), when $x\ge x_s$

25 $$\begin{aligned} \begin{aligned} \beta ^{(\iota)}_{x_{s},x}=\,&(F^{(\iota )}_{x_s+1}-F^{(\iota )}_{x_s})(F^{(\iota )}_{n-x+1}-F^{(\iota )}_{n-x})\\ =&\left( \frac{\lambda ^{n-x+x_s+2}_{\iota }+{\bar{\lambda }}^{n-x+x_s+2}_{\iota }-\lambda ^{n-x+x_s+1}_{\iota }-{\bar{\lambda }}^{n-x+x_s+1}_{\iota }}{(\lambda _{\iota }-\bar{\lambda }_{\iota })^2}-\frac{\lambda ^{n-x+x_s+1}_{\iota }+\bar{\lambda }^{n-x+x_s+1}_{\iota } -\lambda ^{n-x+x_s}_{\iota }-\bar{\lambda }^{n-x+x_s}_{\iota }}{(\lambda _{\iota }-\bar{\lambda }_{\iota })^2}\right) \\&+\left( \frac{\lambda ^{n-x-x_s+1}_{\iota }+\bar{\lambda }^{n-x-x_s+1}_{\iota }-\lambda ^{n-x-x_s}_{\iota }-\bar{\lambda }^{n-x-x_s}_{\iota }}{(\lambda _{\iota }-\bar{\lambda }_{\iota })^2}-\frac{\lambda ^{n-x-x_s}_{\iota }+\bar{\lambda }^{n-x-x_s}_{\iota }-\lambda ^{n-x-x_s-1}_{\iota }-\bar{\lambda }^{n-x-x_s-1}_{\iota }}{(\lambda _{\iota }-\bar{\lambda }_{\iota })^2}\right) \\ =&\left( \frac{(\lambda _{\iota }-1)\lambda ^{n-x+x_s+1}_{\iota }+(\bar{\lambda }_{\iota }-1)\bar{\lambda }^{n-x+x_s+1}_{\iota }-(1-\bar{\lambda }_{\iota })\lambda ^{n-x+x_s+1}_{\iota }-(1-\lambda _{\iota })\bar{\lambda }^{n-x+x_s+1}_{\iota }}{(\lambda _{\iota }-\bar{\lambda }_{\iota })^2}\right) \\&+\left( \frac{(\lambda _{\iota }-1)\lambda ^{n-x-x_s}_{\iota }+(\bar{\lambda }_{\iota }-1)\bar{\lambda }^{n-x-x_s}_{\iota }-(1-\bar{\lambda }_{\iota })\lambda ^{n-x-x_s}_{\iota }-(1-\lambda _{\iota })\bar{\lambda }^{n-x-x_s}_{\iota }}{(\lambda _{\iota }-\bar{\lambda }_{\iota })^2}\right) \\ =\,&\frac{(\lambda _{\iota }+\bar{\lambda }_{\iota }-2)(\lambda ^{n-x+x_s+1}_{\iota }+\bar{\lambda }^{n-x+x_s+1}_{\iota }+\lambda ^{n-x-x_s}_{\iota }+\bar{\lambda }^{n-x-x_s}_{\iota })}{(\lambda _{\iota }-\bar{\lambda }_{\iota })^2}\\ =\,&\frac{(\lambda ^{0.5}_{\iota }-\bar{\lambda }^{0.5}_{\iota })^2}{(\lambda _{\iota }-\bar{\lambda }_{\iota })^2}\left( \lambda ^{n-x+x_s+1}_{\iota }+\bar{\lambda }^{n-x+x_s+1}_{\iota }+\lambda ^{n-x-x_s}_{\iota }+\bar{\lambda }^{n-x-x_s}_{\iota }\right) \\ =\,&({F^{(\iota )}_{0.5}})^2(F^{(\iota )}_{n-(x-x_s)+2}-F^{(\iota )}_{n-(x-x_s)}+F^{(\iota )}_{n-x-x_s+1}-F^{(\iota )}_{n-x-x_s-1}). \end{aligned} \end{aligned}$$

Similarly, when $$x\le x_s$$

26 $$\begin{aligned} \begin{aligned} \beta ^{(\iota)}_{x_{s},x}=&(F^{(\iota )}_{x+1}-F^{(\iota )}_{x})(F^{(\iota )}_{n-x_s+1}-F^{(\iota )}_{n-x_s})\\ =&\left( \frac{\lambda ^{n-x_s+x+2}_{\iota }+\bar{\lambda }^{n-x_s+x+2}_{\iota }-\lambda ^{n-x_s+x+1}_{\iota }-\bar{\lambda }^{n-x_s+x+1}_{\iota }}{(\lambda _{\iota }-\bar{\lambda }_{\iota })^2}-\frac{\lambda ^{n-x_s+x+1}_{\iota }+\bar{\lambda }^{n-x_s+x+1}_{\iota }-\lambda ^{n-x_s+x}_{\iota }-\bar{\lambda }^{n-x_s+x}_{\iota }}{(\lambda _{\iota }-\bar{\lambda }_{\iota })^2}\right) \\&+\left( \frac{\lambda ^{n-x_s-x+1}_{\iota }+\bar{\lambda }^{n-x_s-x+1}_{\iota }-\lambda ^{n-x_s-x}_{\iota }-\bar{\lambda }^{n-x_s-x}_{\iota }}{(\lambda _{\iota }-\bar{\lambda }_{\iota })^2}-\frac{\lambda ^{n-x_s-x}_{\iota }+\bar{\lambda }^{n-x_s-x}_{\iota }-\lambda ^{n-x_s-x-1}_{\iota }-\bar{\lambda }^{n-x_s-x-1}_{\iota }}{(\lambda _{\iota }-\bar{\lambda }_{\iota })^2}\right) \\ =&\left( \frac{(\lambda _{\iota }-1)\lambda ^{n-x_s+x+1}_{\iota }+(\bar{\lambda }_{\iota }-1)\bar{\lambda }^{n-x_s+x+1}_{\iota }-(1-\bar{\lambda }_{\iota })\lambda ^{n-x_s+x+1}_{\iota }-(1-\lambda _{\iota })\bar{\lambda }^{n-x_s+x+1}_{\iota }}{(\lambda _{\iota }-\bar{\lambda }_{\iota })^2}\right) \\&+\left( \frac{(\lambda _{\iota }-1)\lambda ^{n-x_s-x}_{\iota }+(\bar{\lambda }_{\iota }-1)\bar{\lambda }^{n-x_s-x}_{\iota }-(1-\bar{\lambda }_{\iota })\lambda ^{n-x_s-x}_{\iota }-(1-\lambda _{\iota })\bar{\lambda }^{n-x_s-x}_{\iota }}{(\lambda _{\iota }-\bar{\lambda }_{\iota })^2}\right) \\ =&\frac{(\lambda _{\iota }+\bar{\lambda }_{\iota }-2)(\lambda ^{n-x_s+x+1}_{\iota }+\bar{\lambda }^{n-x_s+x+1}_{\iota }+\lambda ^{n-x_s-x}_{\iota }+\bar{\lambda }^{n-x_s-x}_{\iota })}{(\lambda _{\iota }-\bar{\lambda }_{\iota })^2}\\ =&\frac{(\lambda ^{0.5}_{\iota }-\bar{\lambda }^{0.5}_{\iota })^2}{(\lambda _{\iota }-\bar{\lambda }_{\iota })^2}(\lambda ^{n-x_s+x+1}_{\iota }+\bar{\lambda }^{n-x_s+x+1}_{\iota }+\lambda ^{n-x_s-x}_{\iota }+\bar{\lambda }^{n-x_s-x}_{\iota })\\ =&({F^{(\iota )}_{0.5}})^2(F^{(\iota )}_{n-(x_s-x)+2}-F^{(\iota )}_{n-(x_s-x)}+F^{(\iota )}_{n-x_s-x+1}-F^{(\iota )}_{n-x_s-x-1}). \end{aligned} \end{aligned}$$

Combining Eqs. (25) and (26), Eq. (4) is re-expressed by the absolute value functions as

27 $$\begin{aligned} \beta ^{(i)}_{x_{s},x}=({F^{(\iota )}_{0.5}})^2(F^{(\iota )}_{n-|x_s-x|+2} -F^{(\iota )}_{n-|x_s-x|}+F^{(\iota )}_{n-x_s-x+1}-F^{(\iota )}_{n-x_s-x-1}),~~ s = 1,~2, \end{aligned}$$

By Eqs. (21), (27) and (11) in terms of the Chebyshev polynomial of the second class and absolute value function is obtained.

Using Eqs. (19), (21) and (24), the potential formulas (8) and (10) are obtained.

In order to achieve the fast calculation of numerical simulation, we utilize the sixth kind of discrete sine transform to diagonalize the perturbed tridiagonal matrix ${\textbf{A}}_m$^1^.

28 $$\begin{aligned} {\textbf{A}}_{m}= \left( \begin{array}{cccccc} 2+2h &{} -h &{} 0&{} \cdots &{} 0\\ -h &{} 2+2h &{} -h &{} \ddots &{} \vdots \\ 0 &{} \ddots &{} \ddots &{} \ddots &{} 0 \\ \vdots &{} \ddots &{} -h &{} 2+2h &{} -h\\ 0 &{} \cdots &{} 0 &{} -h &{} 2+h \end{array} \right) _{m\times m}, \end{aligned}$$

where $$h = \frac{r}{r_0}$$.

The eigenvectors $\omega _1,\ldots ,\omega _m$ of matrix ${\textbf{A}}_{m}$ are expressed as

29 $$\begin{aligned} \omega _\iota =2+2h-2h\cos \frac{(2\iota -1)\pi }{2m+1}),~~\iota =1,~2,\ldots ,~m, \end{aligned}$$

and the corresponding eigenvectors $\mathbb {\alpha }^{(j)}=(\alpha ^{(j)}_1,\alpha ^{(j)}_2 ,\ldots ,\alpha ^{(j)}_m)^T$ are expressed as

30 $$\begin{aligned} \alpha ^{(j)}_k=\frac{2}{\sqrt{2m+1}}\sin \frac{(2j-1)k\pi }{2m+1}, ~~k=1,~2,\ldots ,~m,~~j=1,~2,\ldots ,~m. \end{aligned}$$

As is known to all, if the orthogonal matrix ${\mathbb {S}}_m^{VI}$ is the sixth kind of discrete sine transform (*DST*-*VI*)^64–68^, where

31 $$\begin{aligned} {\mathbb {S}}_m^{VI}= \frac{2}{\sqrt{2m+1}}\left( \sin \frac{(2j-1)k\pi }{2m+1} \right) _{k,j=1}^m, \end{aligned}$$

then

32 $$\begin{aligned} ({\mathbb {S}}_m^{VI})^{-1}=( {\mathbb {S}}_m^{VI})^T= {\mathbb {S}}_m^{VII}, \end{aligned}$$

where $( {\mathbb {S}}_m^{VI})^T$ is the transpose of the matrix ${\mathbb {S}}_m^{VI}$ and ${\mathbb {S}}_m^{VII}$ is the seventh kind of discrete sine transform (*DST*-*VII*).

The process of realizing the orthogonal diagonalization of matrix ${\textbf{A}}_{m}$ by ${\mathbb {S}}_m^{VI}$ is as follows

33 $$\begin{aligned} ({\mathbb {S}}_m^{VI})^{-1}{\textbf{A}}_{m}({\mathbb {S}}_m^{VI}) =\textrm{diag}(\omega _1,\omega _2,\ldots ,\omega _m), \end{aligned}$$

i.e.,

34 $$\begin{aligned} {\textbf{A}}_{m}=({\mathbb {S}}_m^{VI})\textrm{diag} (\omega _1,\omega _2,\ldots ,\omega _m)({\mathbb {S}}_m^{VI})^{-1}, \end{aligned}$$

where $\omega _\iota$ is given by Eq. (29).

By Kirchhoff’ s law and the node voltage, Tan^1^ gave a matrix equation model as follows

35 $$\begin{aligned} {\textbf{V}}_{v+1} = {\textbf{A}}_{m}{\textbf{V}}_{v} - {\textbf{V}}_{v-1} - r {\textbf{I}}_{v}, \end{aligned}$$

where ${\textbf{A}}_{m}$ in Eq. (28), ${\textbf{V}}_{v}$ and ${\textbf{I}}_{v}$ are vectors of length $m \times 1$, in which $\delta _{k,v}(v =k) = 1$, $\delta _{k,v}(v\ne k) = 0$.

36 $$\begin{aligned} {\textbf{V}}_{v} = [V^{(1)}_{v},V^{(2)}_{v},...V^{(m)}_{v}] ^{T}\ (0 \le v \le n), \end{aligned}$$

37 $$\begin{aligned} I^{(\iota )}_{v}=J(\delta _{x_1,v}-\delta _{x_2,v}). \end{aligned}$$

Since Eq. (35) cannot be directly calculated. Equation (35) is transformed by ${\mathbb {S}}_m^{VI}$ method. The process of transformation is as follows.

38 $$\begin{aligned} ({\mathbb {S}}_m^{VI})^{-1}{\textbf{V}}_{v} =({\mathbb {S}}_m^{VI})^T{\textbf{V}}_{v} = {\textbf{C}}_{v},~~ {\textbf{V}}_{v} = {\mathbb {S}}_m^{VI}{\textbf{C}}_{v}, \end{aligned}$$

where ${\textbf{C}}_{v}$ is also a $m \times 1$ vector

39 $$\begin{aligned} {\textbf{C}}_{v} = [c^{(1)}_{v},c^{(2)}_{v},...,c^{(m)}_{v}]^{T}~~(0 \le v \le n). \end{aligned}$$

### Remark 4

Tan^1^ proposed the node voltage formula of the cobweb network as follows

40 $$\begin{aligned} V^{(y)}_{k} = J\frac{4r}{2m+1}\sum ^{m}_{i=1}\frac{g^{(i)}_{x_{1},x}S_{y_{1},i} -g^{(i)}_{x_{2},x}S_{y_{2},i}}{\lambda ^{n}_{i}+{\bar{\lambda }}^{n}_{i}-2}\sin (y\theta _{i}), \end{aligned}$$

where $g^{(i)}_{x_{1},x}$ is expressed in Eq. (2), $\lambda ^{n}_{i}$ and ${\bar{\lambda }}^{n}_{i}$ is in Eq. (6), $\theta _{i} = \frac{(2i-1)\pi }{(2m+1)}$, $S_{y_k,i} = \sin (y_{k}\theta _{i}),~k=1,~2$.

According to Eqs. (2), (21), (24) and (40), we re-express the node voltage formula by the Chebyshev polynomial of the second class as follows

41 $$\begin{aligned} {\textbf{V}}^{(y)}_{v} = J\frac{4r}{2m+1}\sum ^{m}_{\iota =1}\frac{\mu ^{(\iota )}_{x_{1},x}S_{y_{1},\iota } -\mu ^{(\iota )}_{x_{2},x}S_{y_{2},\iota }}{U^{(\iota )}_{n}-U^{(\iota )}_{n-2}-2}S_{y,\iota }, \end{aligned}$$

where $\mu ^{(\iota )}_{x_{s},x},~s = 1,~2$ is same as Eq. (9), $U^{(\iota )}_{v}$ is same as Eq. (14), and $S_{y_{s},\iota }$ is same as Eq. (12).

According to Eqs. (38), (39) and (41), we can get the analytic formula of ${\textbf{c}}^{(\iota )}_{x}$ as

42 $$\begin{aligned} {\textbf{c}}^{(\iota )}_{x} = \frac{2rJ}{\sqrt{2m+1}}\left( \frac{\mu ^{(\iota )}_{x_{1},x}S_{y_{1},\iota } -\mu ^{(\iota )}_{x_{2},x}S_{y_{2},\iota }}{U^{(\iota )}_{n}-U^{(\iota )}_{n-2}-2}\right) ,~~(0\le x\le n), \end{aligned}$$

Tan^1^ proposed the node voltage formula of the fan network as follows

43 $$\begin{aligned} V^{(y)}_{k} = J\frac{4r}{2m+1}\sum ^{m}_{i=1}\frac{\beta ^{(i)}_{x_{1},x}S_{y_{1},i} -\beta ^{(i)}_{x_{2},x}S_{y_{2},i}}{(t_{i}-2)F^{(i)}_{n+1}}\sin (y\theta _{i}), \end{aligned}$$

where $\beta ^{(i)}_{x_{s},x}$ is expressed in Eq. (4), $F^{(i)}_{k}$ is given in Eq. (5), $t_{i}$ is given in Eq.  (7), $\theta _{i} = \frac{(2i-1)\pi }{(2m+1)}$, $S_{y_k,i} = \sin (y_{k}\theta _{i}),~k=1,~2$.

According to Eqs. (4), (7), (13), (21) and (43), we re-express the node voltage formula by the Chebyshev polynomial of the second class as follows

44 $$\begin{aligned} {\mathcal {V}}^{(y)}_{v} = J\frac{4r}{2m+1}\sum ^{m}_{\iota =1}\frac{\varepsilon ^{(\iota )}_{x_{1},x}S_{y_{1},\iota } -\varepsilon ^{(\iota )}_{x_{2},x}S_{y_{2},\iota }}{(\omega _{\iota }-2)U^{(\iota )}_{n}}S_{y,\iota }, \end{aligned}$$

where $\varepsilon ^{(\iota )}_{x_{s},x},~s = 1,~2$ is same as Eq.  (11), $S_{y_{s},\iota }$ is same as Eq. (12), $\omega _{\iota }$ is same as Eq. (13) and $U^{(\iota )}_{v}$ is same as Eq. (14).

According to Eqs. (38), (39) and (44), we can get the analytic formula of ${{c}}^{(\iota )}_{x}$ as

45 $$\begin{aligned} {{c}}_{x}^{(\iota )} = \frac{2rJ}{\sqrt{2m+1}}\left( \frac{\varepsilon ^{(\iota )}_{x_{1},x}S_{y_{1},\iota } -\varepsilon ^{(\iota )}_{x_{2},x}S_{y_{2},\iota }}{(\omega _{\iota }-2)U^{(\iota )}_{n}}\right) ,~~(0\le x\le n), \end{aligned}$$

## Displaying of some special and interesting potential formulae

According to the obtained resistor network potential formulas (8) and (10) which contain multiple variables, this chapter analyzed the influence of different variables on the resistance network potential formula from two directions, assigned corresponding variables according to the conditions, and drew a three-dimensional dynamic view intuitive display.

### Idiosyncratic potential formulas with the change of current input point and output point position

This section discusses the influence of changes in the position of the input and output points of the current in the resistor network on the potentials, as reflected in the three-dimensional dynamic view.

**Idiosyncratic potential formula 1.** If the current *J* flows in point $d_{1}(x_{1}, y_{1})$ and out of $d_{2}(x_{2}, y_{2})=O_{(0, 0)}$, then a novel potential formula of the cobweb resistor network can be rewritten as

46 $$\begin{aligned} \frac{{\textbf{U}}_{m\times n}(x,y)}{J} = \frac{4r}{2m+1}\sum ^{m}_{\iota =1}\frac{\mu ^{(\iota )}_{x_{1},x}S_{y_{1},\iota }}{U^{(\iota )}_{n}-U^{(\iota )}_{n-2}-2}S_{y,\iota }, \end{aligned}$$

and a novel potential formula of the fan resistor network can be rewritten as

47 $$\begin{aligned} \frac{{\mathcal {U}}_{m\times n}(x,y)}{J} = \frac{4r}{2m+1}\sum ^{m}_{\iota =1}\frac{\varepsilon ^{(\iota )}_{x_{1},x}S_{y_{1},\iota } -\varepsilon ^{(\iota )}_{x_{2},x}S_{y_{2},\iota }}{(\omega _{\iota }-2)U^{(\iota )}_{n}}S_{y,\iota }, \end{aligned}$$

where $\mu ^{(\iota )}_{x_{s},x},~~s = 1,~2$ is defined in Eq.  (9), $\varepsilon ^{(\iota )}_{x_{s},x},~~s = 1,~2,$ is defined in Eq. (11), $U^{(\iota )}_{v}$ is defined in Eq. (14), and $S_{y_{s},\iota }$ is defined in Eq. (12).

Let $m = n = 60,\ J = 10,\ x_{1} = y_{1} = 20,\ x_{2} = y_{2} = 0$, and $r_{0}=r=1$ in Eqs. (46) and (47), respectively. Then a special potential formula of the cobweb resistor network is obtained as follows

48 $$\begin{aligned} \frac{{\textbf{U}}_{60\times 60}(x,y)}{J} = \frac{4}{121}\sum ^{60}_{\iota =1}\frac{\mu ^{(\iota )}_{20,x}S_{20,\iota }}{U^{(\iota )}_{60}-U^{(\iota )}_{58}-2}S_{y,\iota }, \end{aligned}$$

and a special potential formula of the fan resistor network is obtained as follows

49 $$\begin{aligned} \frac{{\mathcal {U}}_{60\times 60}(x,y)}{J} = \frac{4}{121}\sum ^{60}_{\iota =1}\frac{\varepsilon ^{(\iota )}_{20,x}S_{20,\iota }}{(\omega _{\iota }-2)U^{(\iota )}_{60}}S_{y,\iota }, \end{aligned}$$

where

50 $$\begin{aligned} \mu ^{(\iota )}_{20,x} = U^{(\iota )}_{59-|20-x|}+U^{(\iota )}_{|20-x|-1}, \end{aligned}$$

51 $$\begin{aligned} \varepsilon ^{(\iota )}_{20,x}=({U^{(\iota )}_{-0.5}})^2(U^{(\iota )}_{61-|20-x|} -U^{(\iota )}_{59-|20-x|}+U^{(\iota )}_{40-x}-U^{(\iota )}_{38-x}), \end{aligned}$$

52 $$\begin{aligned} \omega _{\iota } = 4 - 2\cos \frac{(2\iota -1)\pi }{121}, \end{aligned}$$

53 $$\begin{aligned} S_{20,\iota } = \sin (\frac{(40\iota -20)\pi }{121}), \end{aligned}$$

54 $$\begin{aligned} U^{(\iota )}_{\nu }= U^{(\iota )}_{\nu }(\cosh \phi _{\iota })= \frac{\sinh (\nu +1)\phi _{\iota }}{\sinh (\phi _{\iota })},~ \cosh \phi _{\iota }=\frac{\omega _{\iota }}{2}, \end{aligned}$$

55 $$\begin{aligned} S_{y,\iota }= \sin \left(\frac{y(2\iota -1)\pi }{121}\right), \end{aligned}$$

$$\begin{aligned} v=61-|20-x|,~59-|20-x|,~|20-x|-1,~40-x,~38-x,~60,~58,~-0.5.~~\iota =1,~2,\ldots ,~60. \end{aligned}$$

**Figure 3:**
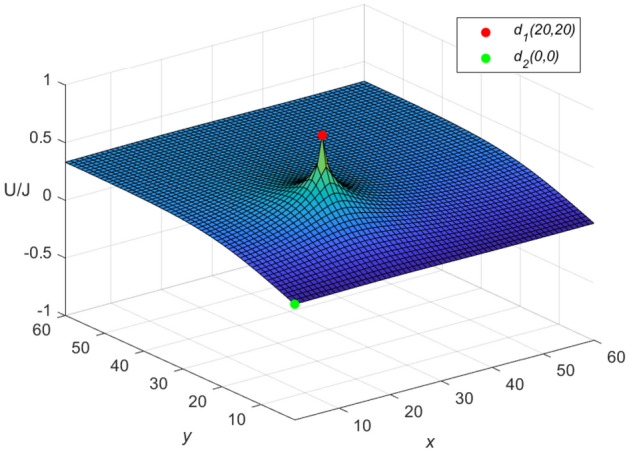
The potential graph for ${\textbf{U}}_{60\times 60}(x,y)/J$ with the cobweb resistor network in Eq. (48).

**Figure 4:**
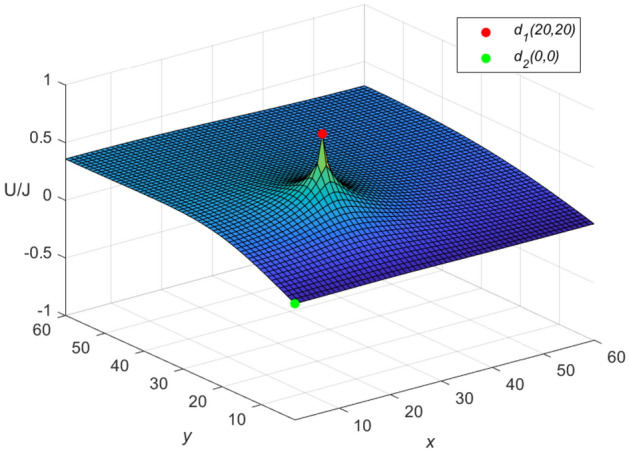
The potential graph for ${\mathcal {U}}_{60\times 60}(x,y)/J$ with the fan resistor network in Eq. (49).

And the three-dimensional dynamic views for the generative process of the potential graph are shown in Figs. 3 and 4, respectively.

**Idiosyncratic potential formula 2.** If the current *J* flows in from point $d_{1}(x_{1}, y_{1})$ and out of $d_{2}(x_{2}, y_{1})$, then a novel potential formula of the cobweb resistor network can be rewritten as

56 $$\begin{aligned} \frac{{\textbf{U}}_{m\times n}(x,y)}{J} = \frac{4r}{2m+1}\sum ^{m}_{\iota =1}\frac{(\mu ^{(\iota )}_{x_{1},x} -\mu ^{(\iota )}_{x_{2},x})S_{y_{1},\iota }}{U^{(\iota )}_{n}-U^{(\iota )}_{n-2}-2}S_{y,\iota }, \end{aligned}$$

and a novel potential formula of the fan resistor network can be rewritten as

57 $$\begin{aligned} \frac{{\mathcal {U}}_{m\times n}(x,y)}{J} = \frac{4r}{2m+1}\sum ^{m}_{\iota =1}\frac{(\varepsilon ^{(\iota )}_{x_{1},x} -\varepsilon ^{(\iota )}_{x_{2},x})S_{y_{1},\iota }}{(\omega _{\iota }-2)U^{(\iota )}_{n}}S_{y,\iota }, \end{aligned}$$

where $\mu ^{(\iota )}_{x_{s},x}$, $\varepsilon ^{(\iota )}_{x_{s},x}$, $S_{y_{s},\iota }$ and $U^{(\iota )}_{v}$ are same as Eqs. (9), (11), (12) and (14), respectively.

Let $m = n = 60$, $J = 10$, $y_{1} = y_{2} = x_{1} = 20$, $x_{2} = 40$, and $r_{0}=r=1$ in Eqs. (56) and (57), respectively. Then an idiosyncratic potential formula of the cobweb resistor network is given by

58 $$\begin{aligned} \frac{{\textbf{U}}_{m\times n}(x,y)}{J} = \frac{4}{121}\sum ^{60}_{\iota =1}\frac{(\mu ^{(\iota )}_{20,x}-\mu ^{(\iota )}_{40,x})S_{20,\iota }}{U^{(\iota )}_{60}-U^{(\iota )}_{58}-2}S_{y,\iota }, \end{aligned}$$

59 $$\begin{aligned} \mu ^{(\iota )}_{40,x} = U^{(\iota )}_{59-|40-x|}+U^{(\iota )}_{|40-x|-1}, \end{aligned}$$

and an idiosyncratic potential formula of the fan resistor network is given by

60 $$\begin{aligned} \frac{{\mathcal {U}}_{m\times n}(x,y)}{J} = \frac{4}{121}\sum ^{60}_{\iota =1}\frac{(\varepsilon ^{(\iota )}_{20,x} -\varepsilon ^{(\iota )}_{40,x})S_{20,\iota }}{(\omega _{\iota }-2)U^{(\iota )}_{60}}S_{y,\iota }, \end{aligned}$$

61 $$\begin{aligned} \varepsilon ^{(\iota )}_{40,x}=({U^{(\iota )}_{-0.5}})^2(U^{(\iota )}_{61-|40-x|} -U^{(\iota )}_{59-|40-x|}+U^{(\iota )}_{20-x}-U^{(\iota )}_{18-x}), \end{aligned}$$

where $\mu ^{(\iota )}_{20,x}$, $\varepsilon ^{(\iota )}_{20,x}$, $\omega _{\iota }$, $S_{20,\iota }$ and $S_{y,\iota }$ are expressed in Eqs. (50), (51), (52), (53) and (55), respectively, with $v=61-|20-x|,~61-|40-x|,~59-|20-x|,~59-|40-x|,~|20-x|-1,~|40-x|-1,~40-x,~38-x,~20-x,~18-x,~60,~58,~-0.5,~~\iota =1,~2,\ldots ,~60$.

**Figure 5:**
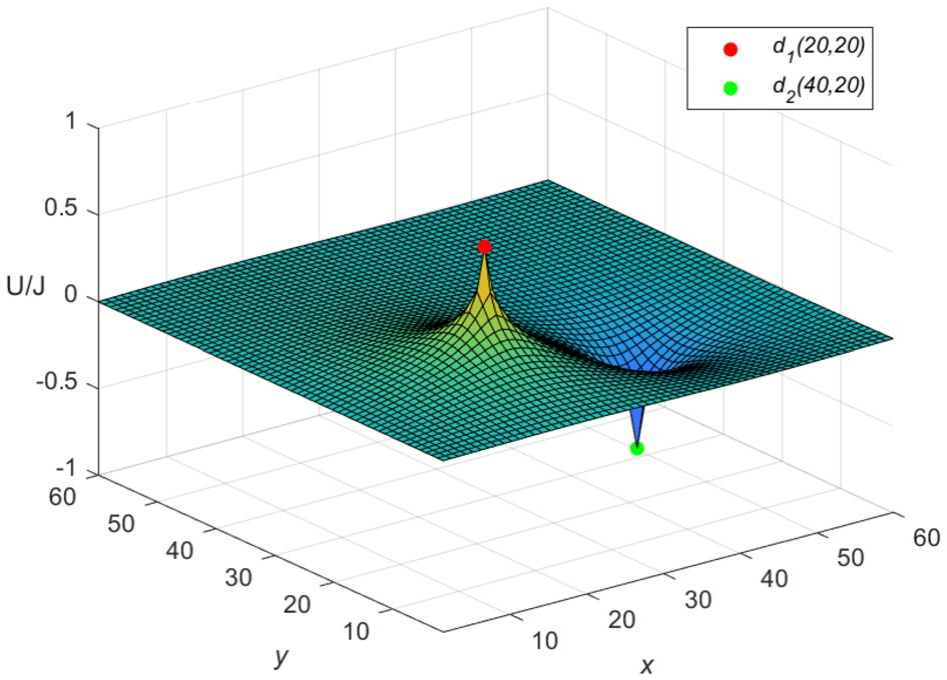
The potential graph for ${\textbf{U}}_{60\times 60}(x,y)/J$ with the cobweb resistor network in Eq. (58).

**Figure 6:**
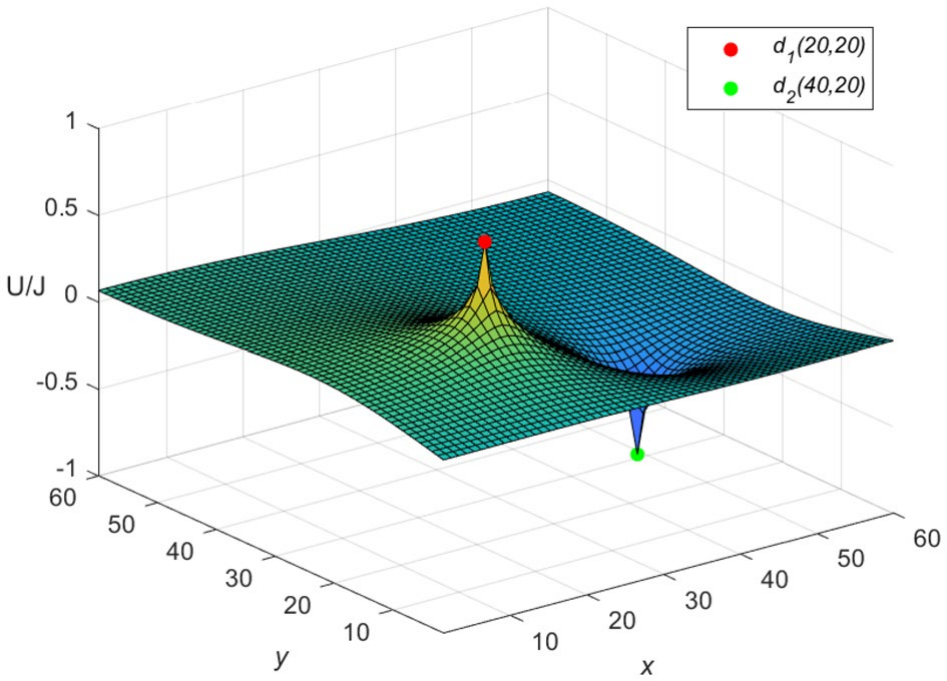
The potential graph for ${\mathcal {U}}_{60\times 60}(x,y)/J$ with the fan resistor network in Eq. (60).

And the three-dimensional dynamic views for the generative process of the potential graph are shown in Figs. 5 and 6 by Matlab.

**Idiosyncratic potential formula 3.** If the current *J*/*h* flows in from point $d_{s}(x_{s}, y_{1}) (s = 1,~2,\ldots ,~h)$ and the current *J* out of $d_{2}(x_{2}, y_{1})$, then a novel potential formula of the cobweb resistor network can be rewritten as

62 $$\begin{aligned} \frac{{\textbf{U}}_{m\times n}(x,y)}{J} = \frac{4r}{2m+1}\sum ^{m}_{\iota =1}\frac{\sum ^{h}_{s=1}\mu ^{(\iota )}_{x_s,x}S_{y_{1},\iota } -\mu ^{(\iota )}_{x_{2},x}S_{y_{2},\iota }}{U^{(\iota )}_{n}-U^{(\iota )}_{n-2}-2}S_{y,\iota }, \end{aligned}$$

and a novel potential formula of the fan resistor network can be rewritten as

63 $$\begin{aligned} \frac{{\mathcal {U}}_{m\times n}(x,y)}{J} = \frac{4r}{2m+1}\sum ^{m}_{\iota =1}\frac{\sum ^{h}_{s=1}\varepsilon ^{(\iota )}_{x_s,x}S_{y_{1},\iota } -\varepsilon ^{(\iota )}_{x_{2},x}S_{y_{2},\iota }}{(\omega _{\iota }-2)U^{(\iota )}_{n}}S_{y,\iota }, \end{aligned}$$

where $\mu ^{(\iota )}_{x_{s},x}$, $\varepsilon ^{(\iota )}_{x_{s},x}$, $S_{y_{s},\iota }$ and $U^{(\iota )}_{v}$ are same as Eqs. (9), (11), (12) and (14), respectively.

Let $m = n = 60,\ J = 10,\ x_{1} = y_{1} = 20,\ x_{2} = y_{2} = 40$, $r_{0}=r=1$, and $h=10$ in Eqs. (62) and (63), respectively. Then an idiosyncratic potential formula of the cobweb resistor network is represented by

64 $$\begin{aligned} \frac{{\textbf{U}}_{m\times n}(x,y)}{J} = \frac{4}{121}\sum ^{60}_{\iota =1}\frac{\sum ^{10}_{s=1}\mu ^{(\iota )}_{x_s,x}S_{20,\iota } -\mu ^{(\iota )}_{40,x}S_{40,\iota }}{U^{(\iota )}_{60}-U^{(\iota )}_{58}-2}S_{y,\iota }, \end{aligned}$$

and an idiosyncratic potential formula of the fan resistor network is represented by

65 $$\begin{aligned} \frac{{\mathcal {U}}_{m\times n}(x,y)}{J} = \frac{4}{121}\sum ^{60}_{\iota =1}\frac{\sum ^{10}_{s=1}\varepsilon ^{(\iota )}_{x_s,x}S_{20,\iota } -\varepsilon ^{(\iota )}_{40,x}S_{40,\iota }}{(\omega _{\iota }-2)U^{(\iota )}_{60}}S_{y,\iota }, \end{aligned}$$

66 $$\begin{aligned} S_{40,\iota } = \sin \left(\frac{(80\iota -40)\pi }{121}\right), \end{aligned}$$

where $\mu ^{(\iota )}_{40,x}$, $\varepsilon ^{(\iota )}_{40,x}$, $\omega _{\iota }$, $S_{20,\iota }$ and $S_{y,\iota }$ are expressed in Eqs. (59), (61), (52), (53) and (55), respectively, with $v=61-|s-x|,~61-|40-x|,~59-|s-x|,~59-|40-x|,~|s-x|-1,~|40-x|-1,~60-s-x,~58-s-x,~20-x,~18-x,~60,~58,~-0.5,~~s=1,~2,\ldots ,~10.~~\iota =1,~2,\ldots ,~60$.

**Figure 7:**
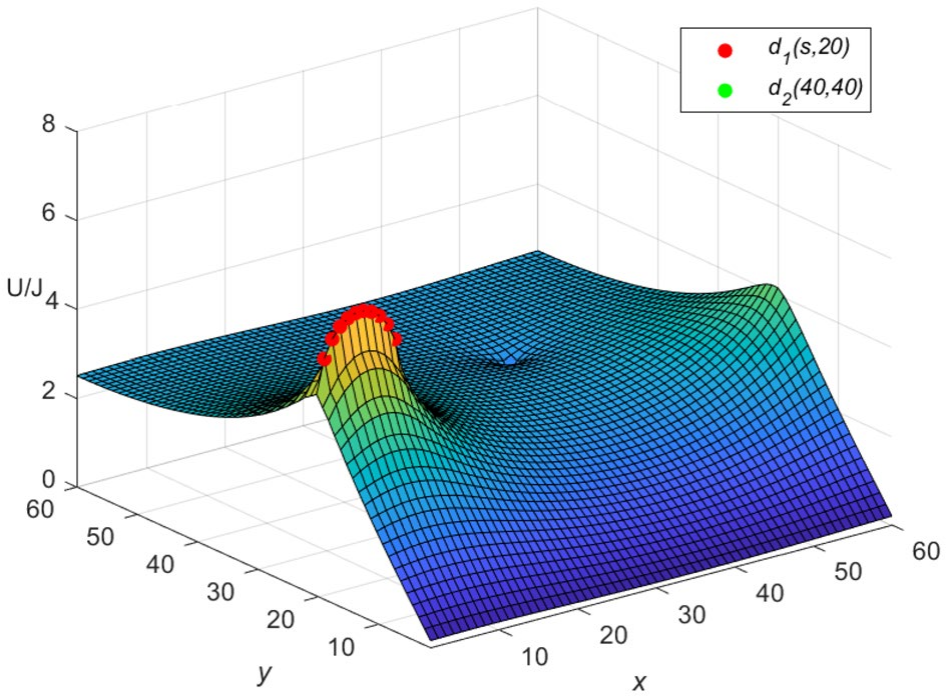
The potential graph for ${\textbf{U}}_{60\times 60}(x,y)/J$ with the cobweb resistor network in Eq. (64).

**Figure 8:**
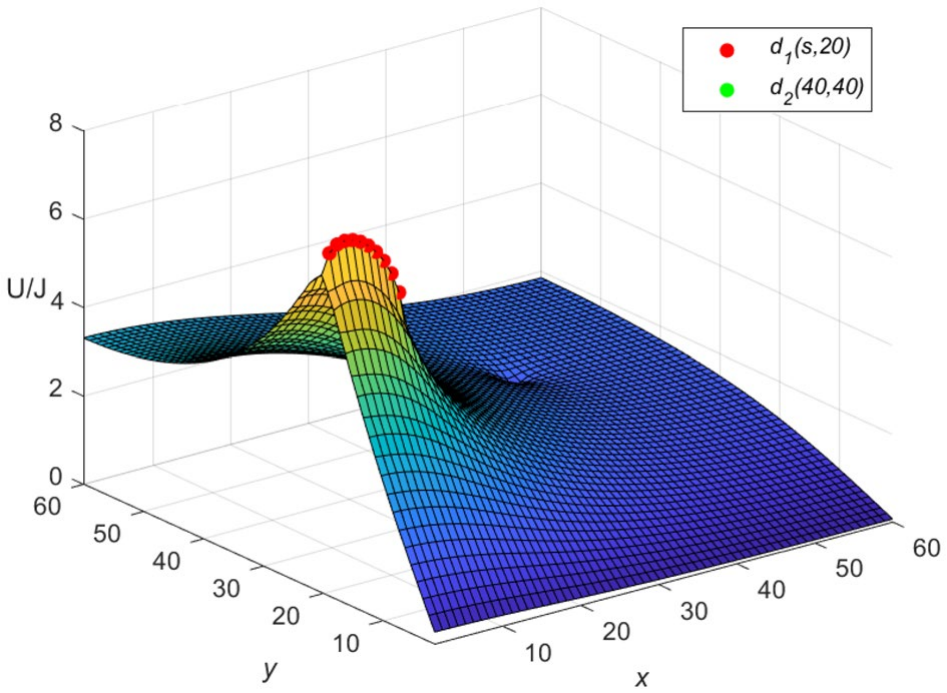
The potential graph for ${\mathcal {U}}_{60\times 60}(x,y)/J$ with the fan resistor network in Eq. (65).

And the three-dimensional dynamic views for the generative process of the potential graph are shown in Figs. 7 and 8 by Matlab.

### Idiosyncratic potential formulas with the change of resistivity *h* ($h=\frac{r}{r_0}$) in resistor network

This section discusses the effect of changes in resistivity *h* in the resistor network on the potential formulas as reflected in the three-dimensional dynamic view.

Let $m = n = 60,\ J = 10,\ x_{1} = y_{1} = 20,\ x_{2} = y_{2} = 40$, and $r=1$ in Eqs. (8) and (10), respectively. Then an idiosyncratic potential formula of the cobweb resistor network is expressed by

67 $$\begin{aligned} \frac{{\textbf{U}}_{60\times 60}(x,y)}{J} = \frac{4}{121}\sum ^{60}_{\iota =1}\frac{\mu ^{(\iota )}_{20,x}S_{20,\iota } -\mu ^{(\iota )}_{40,x}S_{40,\iota }}{U^{(\iota )}_{60}-U^{(\iota )}_{58}-2}S_{y,\iota }, \end{aligned}$$

and an idiosyncratic potential formula of the fan resistor network is expressed by

68 $$\begin{aligned} \frac{{\mathcal {U}}_{60\times 60}(x,y)}{J} = \frac{4}{121}\sum ^{60}_{\iota =1}\frac{\varepsilon ^{(\iota )}_{20,x}S_{20,\iota } -\varepsilon ^{(\iota )}_{40,x}S_{40,\iota }}{(\omega _{\iota }-2)U^{(\iota )}_{60}}S_{y,\iota }, \end{aligned}$$

where $\mu ^{(\iota )}_{20,x}$, $\mu ^{(\iota )}_{40,x}$, $\varepsilon ^{(\iota )}_{20,x}$, $\varepsilon ^{(\iota )}_{40,x}$, $S_{20,\iota }$, $S_{40,\iota }$ and $S_{y,\iota }$ are expressed in Eqs. (50), (59), (51), (61), (53), (66) and (55), respectively.

**Idiosyncratic potential formula 4.** When $r_0=10$, $h=0.1$ is got, as *h* changes, $\omega _{\iota }$ and $\phi _{\iota }$ are obtained as follows, respectively

69 $$\begin{aligned} \omega _{\iota }&= 2.2 - 0.2\cos \frac{(2\iota -1)\pi }{121},\\ \cosh \phi _{\iota }&=1.1-0.1\cos \frac{(2\iota -1)\pi }{121}.\nonumber \end{aligned}$$

**Figure 9:**
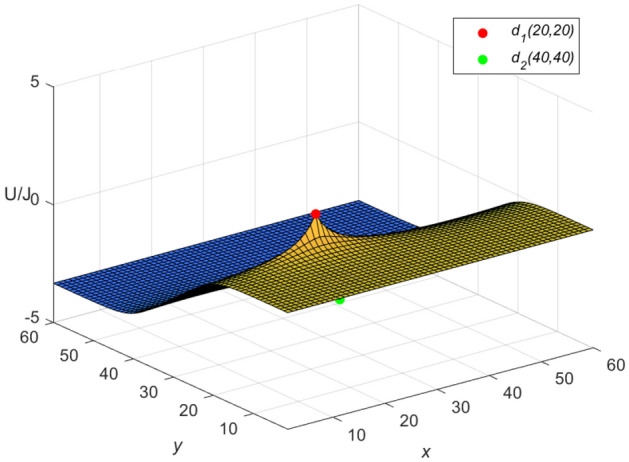
The potential graph for ${\textbf{U}}_{60\times 60}(x,y)/J$ with the cobweb resistor network by Eq. (67).

**Figure 10:**
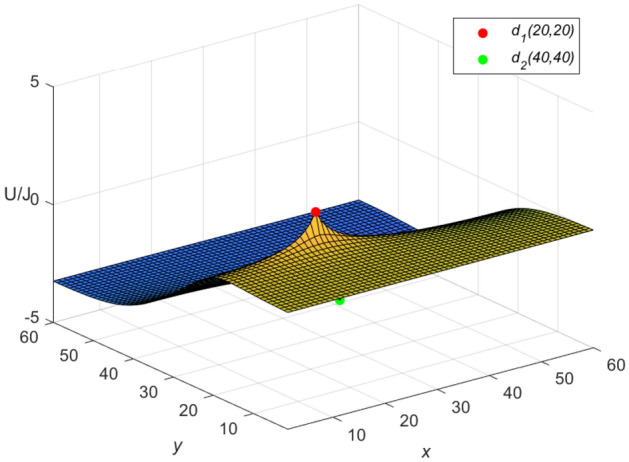
The potential graph for ${\mathcal {U}}_{60\times 60}(x,y)/J$ with the fan resistor network by Eq. (68).

Equation (69) is combined with Eqs. (67) and (68), respectively, and the three-dimensional dynamic views for the generative process of the potential graph are shown in Figs. 9 and 10, respectively.

**Idiosyncratic potential formula 5.** When $r_0=1$, $h=1$ is got, as *h* changes, $\omega _{\iota }$ and $\phi _{\iota }$ are obtained as follows, respectively

70 $$\begin{aligned} \omega _{\iota }&= 4 - 2\cos \frac{(2\iota -1)\pi }{121},\\ \cosh \phi _{\iota }&=2-\cos \frac{(2\iota -1)\pi }{121}.\nonumber \end{aligned}$$

**Figure 11:**
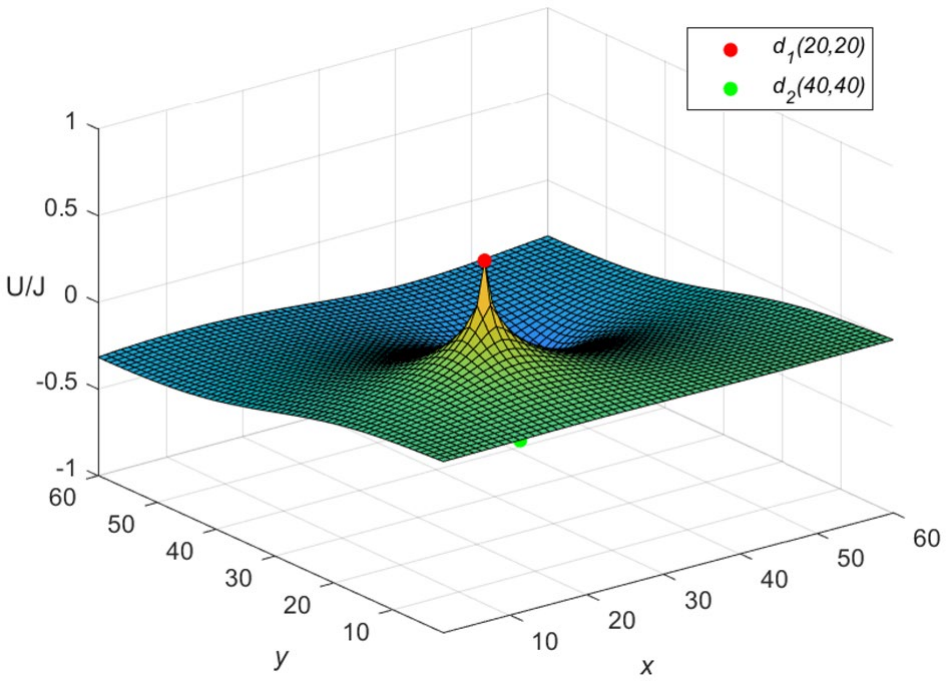
The potential graph for ${\textbf{U}}_{60\times 60}(x,y)/J$ with the cobweb resistor network by Eq. (67).

**Figure 12:**
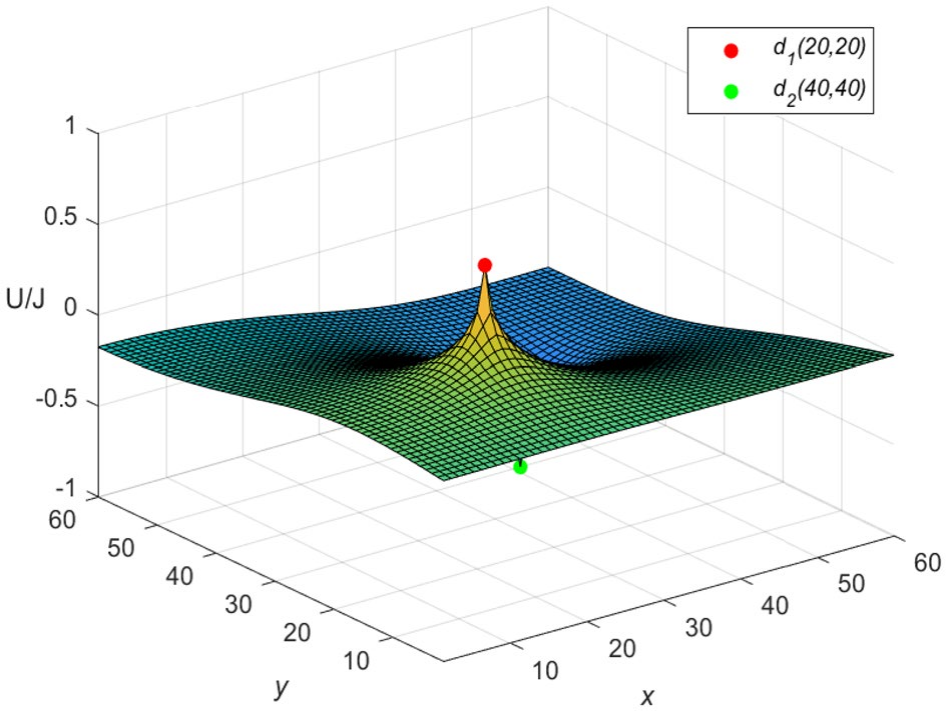
The potential graph for ${\mathcal {U}}_{60\times 60}(x,y)/J$ with the fan resistor network by Eq. (68).

Equation (70) is combined with Eqs. (67) (68), respectively, and the three-dimensional dynamic views for the generative process of the potential graph are shown in Figs. 11 and 12, respectively.

**Idiosyncratic potential formula 6.** When $r_0=0.1$, $h=10$ is got, as *h* changes, $\omega _{\iota }$ and $\phi _{\iota }$ are obtained as follows, respectively

71 $$\begin{aligned} \omega _{\iota }&= 22 - 20\cos \frac{(2\iota -1)\pi }{121},\\ \cosh \phi _{\iota }&=11-10\cos \frac{(2\iota -1)\pi }{121}.\nonumber \end{aligned}$$

**Figure 13:**
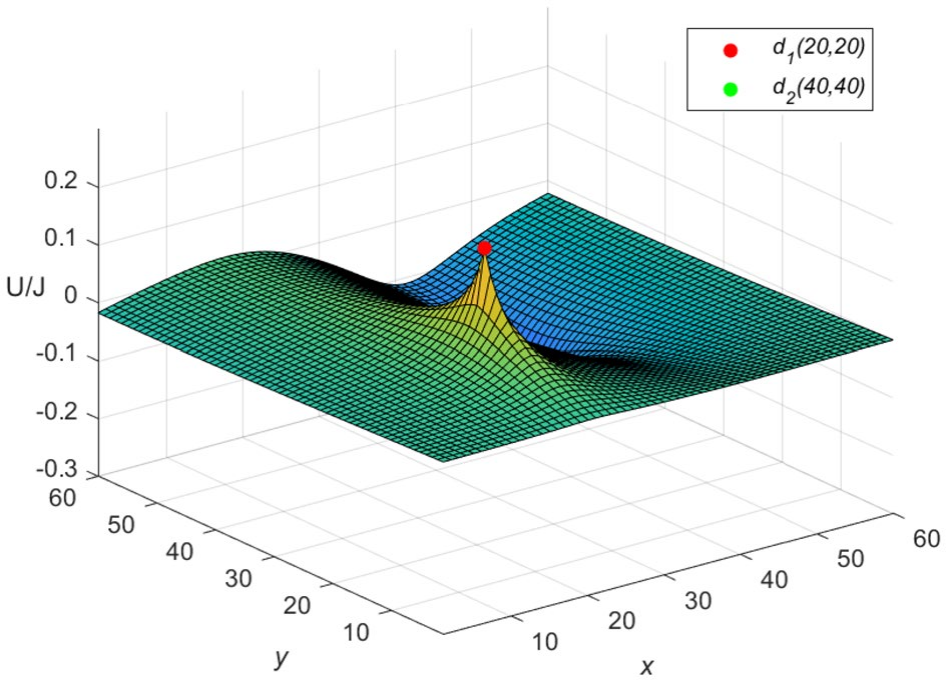
The potential graph for ${\textbf{U}}_{60\times 60}(x,y)/J$ with the cobweb resistor network by Eq. (67).

**Figure 14:**
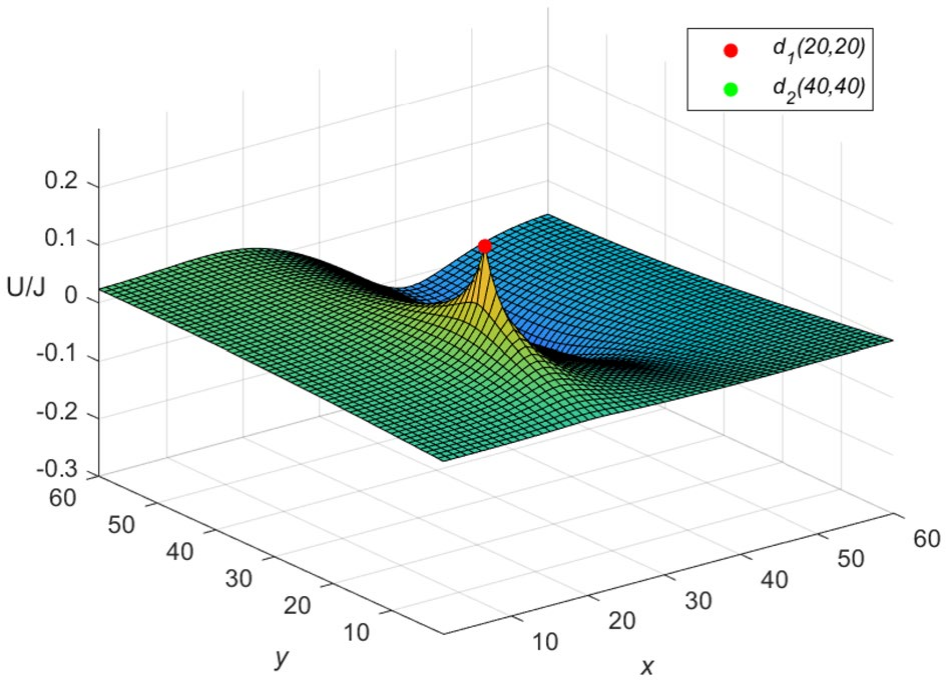
The potential graph for ${\mathcal {U}}_{60\times 60}(x,y)/J$ with the fan resistor network by Eq. (68).

Equation (71) is combined with Eqs. (67) and (68), respectively, and the three-dimensional dynamic views for the generative process of the potential graph are shown in Figs. 13 and 14, respectively.

## Numerical algorithms for computing potential

Combining the *DST*-*VI* and Eqs. (30), (31), (32), (33), (34), this chapter provides two numerical algorithms to achieve fast calculation of large-scale potential for the resistor network. The numerical algorithm obtains similar results to the potential formulas (8) and (10).
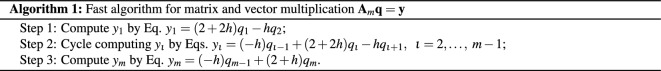

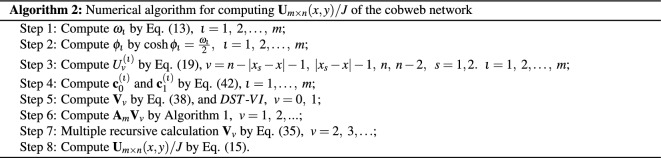

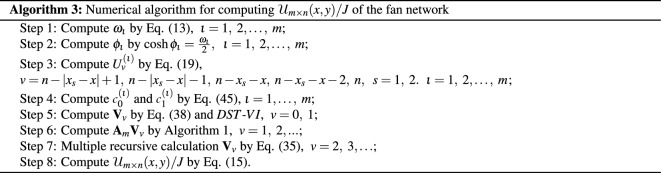


### Remark 5

As is well-known, the Algorithm 1 is a tridiagonal matrix-vector multiplication, which the computational complexity is *O*(*n*). Moreover, one *DST*-*VI* needs $2n\log _2 n+ O(n)$ real arithmetic operations^68^. So the Algorithm 2 composed of Algorithm 1 and two *DST*-*VI*, and it’s computational complexity is $4n\log _2 n+ O(n)$. Analogous, the computational complexity of Algorithms 3 is also $4n\log _2 n+ O(n)$. According to the above Algorithms 2 and Algorithms 3, two instances are used to display the iterative effect of large-scale data graphically in the following.

**Figure 15:**
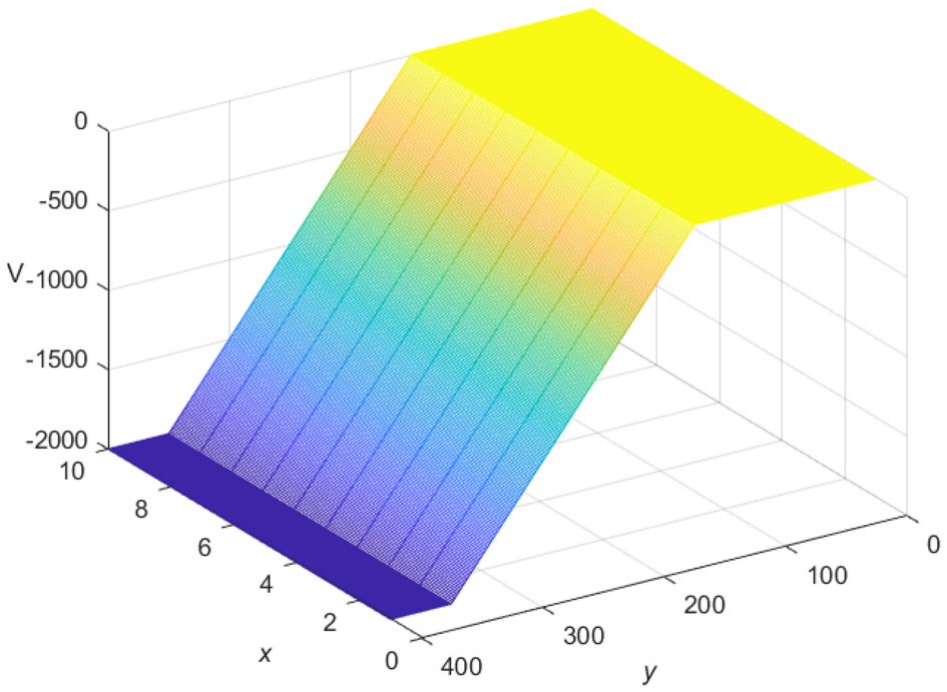
A 3D image display for the fast Algorithm 2 of ${\textbf{U}}_{400\times 10}(x,y)/J$ on the cobweb resistor network.

**Figure 16:**
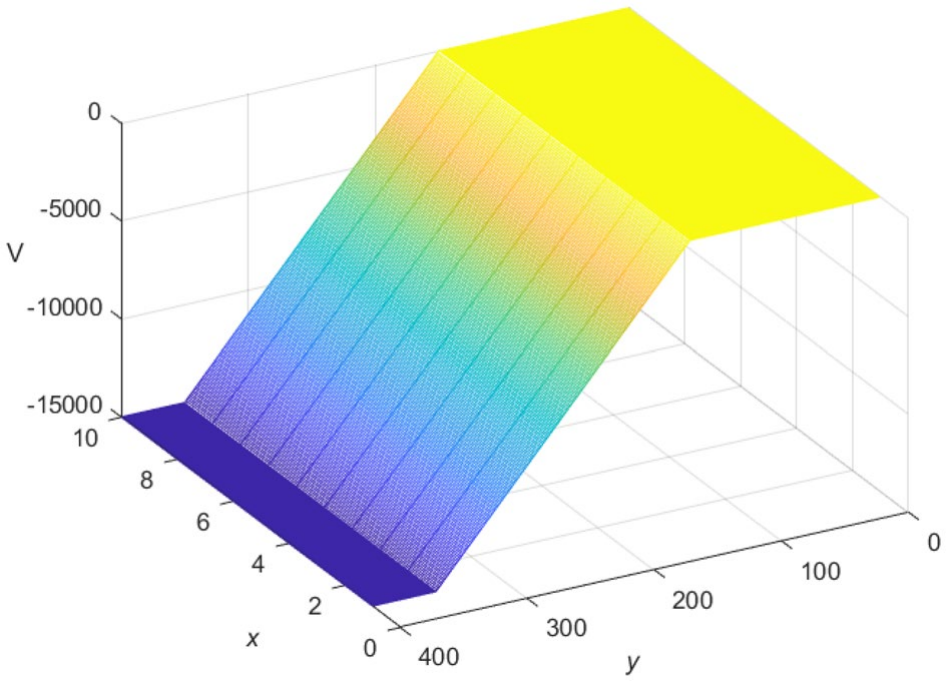
A 3D image display for the fast Algorithm 3 of ${\mathcal {U}}_{400\times 10}(x,y)/J$ on the fan resistor network.

Let $m = 400$ and $n = 10$, the current *J* flows from the $d_{1}(x_{1},y_{1})$ point, $x_{1} =3, y_{1} = 150$, and out from the $d_{2}(x_{2},y_{2})$ point, $x_{2} =7,y_{2} = 350$. $r = 1$, $r_{0} = 100$, and $J = 10$. The fast algorithm of cobweb resistor network is shown in Fig. 15, and the fast algorithm of fan resistor network is shown in Fig. 16.

## Efficiency of calculation method

On the $m \times n$ scale resistor network models, $(x_{1},y_{1})$ refers to the input point of the current and $(x_{2},y_{2})$ refers to the output point of the current. We give a comparison of calculation efficiency for the calculating potential in three different methods. “Time” is the total CPU time in seconds, $t_1$, $t_2$ and $t_3$ denote CPU times of the potential computed by formulas (1), (3), formulas (8), (10) and Algorithm 2, 3, respectively.

The experiment is completed under the environmental conditions of CPU model AMD R9-5900HX, CPU frequency 3.30 GHz, and Matlab version is R2020b. $``m \times n"$ is the number of nodes in the resistor network., $``-"$ denotes the operation time more than 1200s or beyond the memory limit of Matlab.

**Table 1:** The comparison of calculation efficiency for potential formulas (1) and (8).

$m \times n$	$(x_{1},y_{1})$	$(x_{2},y_{2})$	$r/r_0$	$t_1$	$t_2$
$100 \times 100$	(40 , 40)	(80 , 80)	1	0.139	0.029
$200 \times 200$	(40 , 40)	(180 , 180)	1	0.923	0.121
$300 \times 300$	(40 , 40)	(180 , 180)	1	2.817	0.340
$400 \times 400$	(40 , 40)	(180 , 180)	1	7.685	1.649
$800 \times 100$	(40 , 40)	(80 , 80)	1	6.652	0.874
$800 \times 200$	(40 , 40)	(180 , 180)	1	15.269	3.510

**Table 2:** The comparison of calculation efficiency for potential formulas (3) and (10).

$m \times n$	$(x_{1},y_{1})$	$(x_{2},y_{2})$	$r/r_0$	$t_1$	$t_2$
$100 \times 100$	(40 , 40)	(80 , 80)	1	0.271	0.045
$200 \times 200$	(40 , 40)	(180 , 180)	1	1.667	0.244
$300 \times 300$	(40 , 40)	(180 , 180)	1	–	0.631
$400 \times 400$	(40 , 40)	(180 , 180)	1	–	2.964
$800 \times 100$	(40 , 40)	(80 , 80)	1	13.210	1.546
$800 \times 200$	(40 , 40)	(180 , 180)	1	30.351	5.925

**Table 3:** The comparison of calculation efficiency for potential formulas (1) and (8).

$m \times n$	$(x_{1},y_{1})$	$(x_{2},y_{2})$	$r/r_0$	$t_1$	$t_2$
$400 \times 400$	(40 , 40)	(180 , 180)	0.1	7.626	1.760
$500 \times 500$	(40 , 40)	(180 , 180)	0.1	14.666	3.001
$600 \times 600$	(40 , 40)	(180 , 180)	0.1	25.365	4.932
$1100 \times 1100$	(40 , 40)	(180 , 180)	0.1	159.767	34.444
$1000 \times 400$	(40 , 40)	(180 , 180)	0.1	47.323	9.187
$1000 \times 500$	(40 , 40)	(180 , 180)	0.1	59.539	11.613

**Table 4:** The comparison of calculation efficiency for potential formulas (3) and (10).

$m \times n$	$(x_{1},y_{1})$	$(x_{2},y_{2})$	$r/r_0$	$t_1$	$t_2$
$400 \times 400$	(40 , 40)	(180 , 180)	0.1	14.962	3.016
$500 \times 500$	(40 , 40)	(180 , 180)	0.1	28.453	5.325
$600 \times 600$	(40 , 40)	(180 , 180)	0.1	–	9.052
$1100 \times 1100$	(40 , 40)	(180 , 180)	0.1	–	63.093
$1000 \times 400$	(40 , 40)	(180 , 180)	0.1	91.827	16.498
$1000 \times 500$	(40 , 40)	(180 , 180)	0.1	118.262	20.461

**Table 5:** The comparison of calculation efficiency for potential formulas (1) and (8).

$m \times n$	$(x_{1},y_{1})$	$(x_{2},y_{2})$	$r/r_0$	$t_1$	$t_2$
$1000 \times 1000$	(40 , 40)	(580 , 580)	0.01	120.787	25.788
$1500 \times 1500$	(40 , 40)	(580 , 580)	0.01	405.983	92.694
$1800 \times 1800$	(40 , 40)	(580 , 580)	0.01	700.525	159.449
$2000 \times 2000$	(40 , 40)	(580 , 580)	0.01	958.713	217.022
$1500 \times 1000$	(40 , 40)	(580 , 580)	0.01	270.714	61.578
$2000 \times 1000$	(40 , 40)	(580 , 580)	0.01	480.070	110.790

**Table 6:** The comparison of calculation efficiency for potential formulas (3) and (10).

$m \times n$	$(x_{1},y_{1})$	$(x_{2},y_{2})$	$r/r_0$	$t_1$	$t_2$
$1000 \times 1000$	(40 , 40)	(580 , 580)	0.01	234.791	46.840
$1500 \times 1500$	(40 , 40)	(580 , 580)	0.01	792.929	171.828
$1800 \times 1800$	(40 , 40)	(580 , 580)	0.01	1373.612	308.328
$2000 \times 2000$	(40 , 40)	(580 , 580)	0.01	–	410.130
$1500 \times 1000$	(40 , 40)	(580 , 580)	0.01	528.783	114.033
$2000 \times 1000$	(40 , 40)	(580 , 580)	0.01	943.748	208.219

**Table 7:** Comparison of the efficiency for potential formulas (1), (8) and Algorithm 2.

$m \times n$	$(x_{1},y_{1})$	$(x_{2},y_{2})$	$r/r_0$	$t_1$	$t_2$	$t_3$
$100 \times 10$	(3 , 30)	(5 , 50)	0.01	0.031	0.013	0.013
$1000 \times 10$	(3 , 300)	(5 , 500)	0.01	1.144	0.213	0.033
$10000 \times 10$	(3 , 3000)	(5 , 5000)	0.01	105.350	11.588	1.098
$20000 \times 10$	(3 , 3000)	(5 , 5000)	0.01	474.890	93.546	4.156
$30000 \times 10$	(3 , 3000)	(5 , 5000)	0.01	1043.302	204.098	8.369
$40000 \times 10$	(3 , 3000)	(5 , 5000)	0.01	1857.076	358.305	19.710

**Table 8:** Comparison of the efficiency for potential formulas (3), (10) and Algorithm 3.

$m \times n$	$(x_{1},y_{1})$	$(x_{2},y_{2})$	$r/r_0$	$t_1$	$t_2$	$t_3$
$100 \times 10$	(3 , 30)	(5 , 50)	0.01	0.035	0.017	0.013
$1000 \times 10$	(3 , 300)	(5 , 500)	0.01	1.188	0.388	0.050
$10000 \times 10$	(3 , 3000)	(5 , 5000)	0.01	106.246	21.425	1.014
$20000 \times 10$	(3 , 3000)	(5 , 5000)	0.01	468.767	175.736	3.465
$30000 \times 10$	(3 , 3000)	(5 , 5000)	0.01	1041.555	365.012	7.998
$40000 \times 10$	(3 , 3000)	(5 , 5000)	0.01	1858.145	637.289	19.353

### Remark 6

Tables 1, 3, 5 show the calculation time of cobweb resistor network with different square and rectangular sizes at different resistivity. The optimized potential formula (8) has faster operation speed.

### Remark 7

Tables 2, 4, 6 show the calculation time of fan resistor network with different square and rectangular sizes at different resistivity. The optimized potential formula (10) has faster operation speed.

### Remark 8

Table 7 shows the efficiency of the potential formula (1), formula (8) and the Algorithm 2 for calculating the potential. Algorithm 2 not only realizes large-scale calculation, but also has shorter calculation time in calculating cobweb resistor network.

### Remark 9

Table 8 shows the efficiency of the potential formula (3), formula (10) and the Algorithm 3 for calculating the potential. Algorithm 3 not only realizes large-scale calculation, but also has shorter calculation time in calculating fan resistor network.

## Conclusion

In this paper, based on the *RT*-*V* method, the accurate potential formulas of the $m \times n$ cobweb resistor network and the $m \times n$ fan resistor network^1^ are improved. The potential formula is represented by the Chebyshev polynomial of the second class and the tridiagonal matrix is diagonalized by the *DST*-*VI* method, which realizes the high efficiency of the numerical simulation of the potential formula. The changes of variables in the potential formula are analyzed, and the corresponding three-dimensional view is drawn to show the influence of variable changes on the image. Then we design a fast algorithm for the resistor network potential to achieve fast calculation in the case of large-scale resistor networks. Finally, we show the calculation time of different calculation methods under different scale resistor networks, and the comparison shows the efficiency of the improved numerical simulation calculation.

## Data Availability

All data generated or analysed during this study are included in this article and its supplementary information files.
